# Supplementary material for: FLEXIQuant-LF to quantify protein modification extent in label-free proteomics data
Source: eLife. 2020 Dec 7;9:e58783. doi: 10.7554/eLife.58783 (PMC7721442; doi:10.7554/eLife.58783)
Supplement: Transparent reporting form [file elife-58783-transrepform.pdf]

eLIFE
Westbrook Centre, Milton Road
Cambridge CB4 1YG
UK
P 01223 855340
W elifesciences.org
T @elife

eLife's transparent reporting form

We encourage authors to provide detailed information *within their submission* to facilitate the interpretation and replication of experiments. Authors can upload supporting documentation to indicate the use of appropriate reporting guidelines for health-related research (see EQUATOR Network), life science research (see the BioSharing Information Resource), or the ARRIVE guidelines for reporting work involving animal research. Where applicable, authors should refer to any relevant reporting standards documents in this form.

If you have any questions, please consult our Journal Policies and/or contact us: editorial@elifesciences.org.

Sample-size estimation

- You should state whether an appropriate sample size was computed when the study was being designed
- You should state the statistical method of sample size computation and any required assumptions
- If no explicit power analysis was used, you should describe how you decided what sample (replicate) size (number) to use

Please outline where this information can be found within the submission (e.g., sections or figure legends), or explain why this information doesn't apply to your submission:

Timepoints and replicate numbers were decided based on experience of the techniques performed and practical considerations for mass spectrometry analysis (see SWATH sample acquisition in the Methods section).

Replicates

- You should report how often each experiment was performed
- You should include a definition of biological versus technical replication
- The data obtained should be provided and sufficient information should be provided to indicate the number of independent biological and/or technical replicates
- If you encountered any outliers, you should describe how these were handled
- Criteria for exclusion/inclusion of data should be clearly stated
- High-throughput sequence data should be uploaded before submission, with a private link for reviewers provided (these are available from both GEO and ArrayExpress)

Please outline where this information can be found within the submission (e.g., sections or figure legends), or explain why this information doesn't apply to your submission:

Mass spectrometry data were acquired in triplicates (technical replicates) for each timepoint. Single replicates were removed at time points 4h and 10h due to outlier (low) numbers of identified/quantified proteins.
Additional independent publicly available data were selected to have three technical replicates were acquired for the analysis.
The software tool FLEXIQuant-LF was tested in repeats of n=1000 for increasing numbers of internal repeats of RANSAC (see Reproducibility in the Methods section)

Statistical reporting

- Statistical analysis methods should be described and justified
- Raw data should be presented in figures whenever informative to do so (typically when N per group is less than 10)
- For each experiment, you should identify the statistical tests used, exact values of N, definitions of center, methods of multiple test correction, and dispersion and precision measures (e.g., mean, median, SD, SEM, confidence intervals; and, for the major substantive results, a measure of effect size (e.g., Pearson's r, Cohen's d)
- Report exact p-values wherever possible alongside the summary statistics and 95% confidence intervals. These should be reported for all key questions and not only when the p-value is less than 0.05.

Please outline where this information can be found within the submission (e.g., sections or figure legends), or explain why this information doesn't apply to your submission:

General information on the algorithm and use of dispersion and precision measures is given at the beginning of the Methods section. Replicates of time points were collapsed to single values (median) to account for outliers. RANSAC regression selection is based on optimal r2 value in 30 repeats.
Quality and error estimation analysis was performed on independent publicly available data used as a basis for simulated peptide intensities. Replicates were treated separately.

(For large datasets, or papers with a very large number of statistical tests, you may upload a single table file with tests, Ns, etc., with reference to sections in the manuscript.)

Group allocation

- Indicate how samples were allocated into experimental groups (in the case of clinical studies, please specify allocation to treatment method); if randomization was used, please also state if restricted randomization was applied
- Indicate if masking was used during group allocation, data collection and/or data analysis

Please outline where this information can be found within the submission (e.g., sections or figure legends), or explain why this information doesn't apply to your submission:

Experimental groups are defined by measured timepoints. No additional allocation into groups was performed.

Additional data files ("source data")

- We encourage you to upload relevant additional data files, such as numerical data that are represented as a graph in a figure, or as a summary table
- Where provided, these should be in the most useful format, and they can be uploaded as "Source data" files linked to a main figure or table
- Include model definition files including the full list of parameters used
- Include code used for data analysis (e.g., R, MatLab)
- Avoid stating that data files are "available upon request"

Please indicate the figures or tables for which source data files have been provided:

eLife Sciences Publications, Ltd is a limited liability non-profit non-stock corporation incorporated in the State of Delaware, USA, with company number 5030732, and is registered in the UK with company number FC030576 and branch number BR015634 at the address Westbrook Centre, Milton Road Cambridge CB4 1YG, UK | March 2019

Raw data and proteomics search results are available through ProteomeXchange with identifier PXD018411 (see Data and Software availability). Raw data and result files from the additional publicly available independent dataset are available through ProteomeXchange with identifier PXD005573/ The software code is made available on GitHub, https://github.com/SteenOmicsLab/FLEXIQuantLF. Processed data are provided as tables and supplementary tables as part of the manuscript.
